# Advances and perspectives of PARP inhibitors

**DOI:** 10.1186/s40164-019-0154-9

**Published:** 2019-11-11

**Authors:** Ming Yi, Bing Dong, Shuang Qin, Qian Chu, Kongming Wu, Suxia Luo

**Affiliations:** 1 Department of Oncology, Tongji Hospital, Tongji Medical College, Huazhong University of Science and Technology, Wuhan, 430030 China; 2 Department of Molecular Pathology, The Affiliated Cancer Hospital, Zhengzhou University & Henan Cancer Hospital, Zhengzhou, 450008 China; 3 Department of Medical Oncology, The Affiliated Cancer Hospital, Zhengzhou University & Henan Cancer Hospital, Zhengzhou, 450008 China

**Keywords:** PARPi, DNA repair, Synthetic lethal, Combination therapy, Immune checkpoint inhibitor

## Abstract

DNA damage repair deficiency leads to the increased risk of genome instability and oncogenic transformation. In the meanwhile, this deficiency could be exploited for cancer treatment by inducing excessive genome instability and catastrophic DNA damage. Continuous DNA replication in cancer cells leads to higher demand of DNA repair components. Due to the oncogenic loss of some DNA repair effectors (e.g. BRCA) and incomplete DNA repair repertoire, some cancer cells are addicted to certain DNA repair pathways such as Poly (ADP-ribose) polymerase (PARP)-related single-strand break repair pathway. The interaction between BRCA and PARP is a form of synthetic lethal effect which means the simultaneously functional loss of two genes lead to cell death, while defect in any single gene has a slight effect on cell viability. Based on synthetic lethal theory, Poly (ADP-ribose) polymerase inhibitor (PARPi) was developed aiming to selectively target cancer cells harboring BRCA1/2 mutations. Recently, a growing body of evidence indicated that a broader population of patients could benefit from PARPi therapy far beyond those with germline BRCA1/2 mutated tumors. Numerous biomarkers including homologous recombination deficiency and high level of replication pressure also herald high sensitivity to PARPi treatment. Besides, a series of studies indicated that PARPi-involved combination therapy such as PARPi with additional chemotherapy therapy, immune checkpoint inhibitor, as well as targeted agent had a great advantage in overcoming PARPi resistance and enhancing PARPi efficacy. In this review, we summarized the advances of PARPi in clinical application. Besides, we highlighted multiple promising PARPi-based combination strategies in preclinical and clinical studies.

## Introduction

As the hallmark of cancers, genome instability participates in the initiation and progression of cancers by inducing the generation of mutations and neoantigens [1–4]. Genome instability is closely related with inadequate repertoire of DNA repair pathways [5, 6]. For sustaining cell viability, cancer cells highly depend on some specific DNA damage repair pathways to control DNA damage events in a low level [7]. Thus, these essential repair pathways are ideal targets for cancer treatment [8, 9].

In normal cells, DNA damages could be detected and repaired by DNA single-strand break (SSB) repair pathways or double-strand break (DSB) repair pathways [10, 11]. SSB repair pathways include mismatch repair (MMR), nucleotide excision repair (NER), and base excision repair (BER), while DSB repair pathways include homologous recombination (HR) and nonhomologous end joining (NHEJ) [12–14]. Compared with SSB, DSB is a more cytotoxic form of DNA damage [15]. When replicated sister chromatid and key molecules in HR pathway such as BRCA1/2 are available, cell could faithfully repair DSB damages by HR [16]. In the absence of template DNA or intact HR pathway, NHEJ pathway is adopted to repair DSB damages [17]. However, NHEJ is a rapid as well as error-prone repair pathway by direct ligation [18]. Due to the low-fidelity, NHEJ often produces plenty of chromosomal rearrangements and these unsustainable DNA damages are harmful to cell viability [19]. For HR deficient tumor cells, intact SSB repair pathways is the vital prerequisites for cell survival [20].

Based on synthetic lethality theory, simultaneously blocking SSB repair and HR repair pathways could severely inhibit cell survival [20]. Therefore, as the core component of SSB, Poly (ADP-ribose) polymerase (PARP) is an ideal treatment target for HR deficient cancers [21]. Initially, it was found that PARP inhibitor (PARPi) could effectively kill BRCA1/2 mutated tumor cells [22, 23]. Later, it was noticed that some non-BRCA1/2 mutated HR deficient tumors were sensitive to PARPi treatment as well [24]. PARPi not only inhibits the catalytic activity of PARP, but also traps PARP on damaged DNA site [25, 26]. The persistent PARP-DNA chain complex leads to the stalling of DNA replication fork [27]. Then, DNA replication fork collapses and generates DSB [27]. Due to the difference of HR status between normal cells and cancer cells, PARPi-induced DSB could be repaired by HR pathway in normal cells while the DSB is repaired by NHEJ pathway in cancer cells [27]. As a result, tumor cells harboring HR deficiency are more sensitive to PARPi therapy than normal cells (e.g. over 1000 times in BRCA1/2 mutated tumor cells) [27]. In theory, a wider group of patients could benefit from PARPi treatment beyond germline BRCA1/2 mutated (gBRCAm) patients.

## The structure and function of PARP

**Fig. 1:**
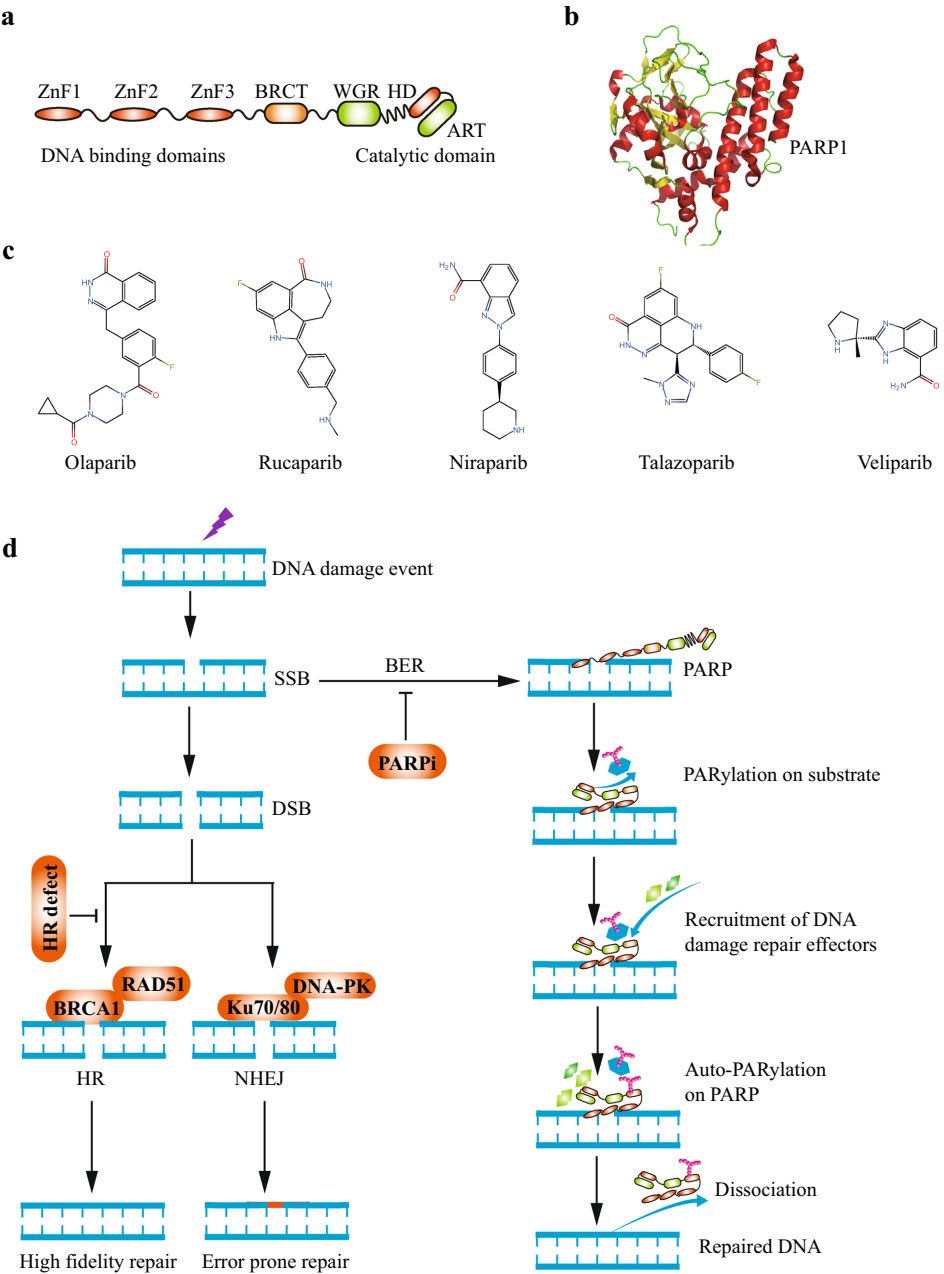
PARP and PARPis. **a** The structure schematic of PARP molecule. There are six main domains of PARP1 which include three zinc finger-related domains (DNA binding domains), one BRCA1 C-terminus domain (auto-modification domain), one tryptophan-/glycine-/arginine-rich domain (WGR domain), and one catalytic domain. The catalytic domain of PARP1 consists of two subdomains: one helical domain (HD) and one ADP-ribosyltransferase catalytic domain (ART). **b** Three-dimensional structure of PARP1 molecule. The above structures were presented by PyMOL Molecular Graphics System (PDB ID: 5XST [104]). **c** The chemistry structural formula of PARPis. **d** The function of PARP and synthetic lethal interaction between PARP and HR. Once DNA SSB emerges, PARP1 could recognizes and interacts with SSB by its zinc finger-related domains. After PARP1 binding to damaged DNA chains, the auto-inhibitory function of HD is abrogated and the catalytic function of ART is activated. This catalytic activity leads to the generation of PAR chains on a series of target proteins which promotes the recruitment of DNA repair effectors and chromatin remodeling. Then the auto-PARylation on PARP1 protein causes the dissociation of PARP1 from DNA chains and restores the auto-inhibitory status of PARP1. For HR deficient tumor cells, active SSB repair pathways is the vital prerequisites for cell survival. After PARPi treatment, NHEJ is used to repair PARPi-introduced DSB. However, NHEJ is a rapid as well as error-prone repair pathway by direct ligation. Due to the low-fidelity, NHEJ often produces plenty of chromosomal rearrangements and these unsustainable DNA damages are harmful to cell viability

Poly (ADP-ribose) polymerase (PARP) family of enzymes participate in various cellular processes via covalently adding poly (ADP-ribose) chains onto target molecules (also termed as PARylation) [28]. Among all proteins belonging to PARP family, PARP1 is mostly correlated with DNA damage repair which generates nearly 90% of poly (ADP-ribose) chains after DNA damage event [29]. There are six main domains of PARP1 which include three zinc finger-related domains (DNA binding domains), one BRCA1 C-terminus domain (auto-modification domain), one tryptophan-/glycine-/arginine-rich domain (WGR domain), and one catalytic domain (Fig. 1) [30]. The catalytic domain of PARP1 consists of two subdomains: one helical domain (HD) and one ADP-ribosyltransferase catalytic domain (ART) [30]. In the non-DNA binding status, HD inhibits the binding between PARP1 and its cofactor β-nicotinamide adenine dinucleotide (β-NAD) in ART [30, 31]. Once DNA SSB emerges, PARP1 could recognize and interact with SSB by its zinc finger-related domains [32]. After PARP1 binding to damaged DNA chains, the auto-inhibitory function of HD is abrogated and the catalytic function of ART is activated [32]. This catalytic activity leads to the generation of PAR chains on a series of target proteins which promotes the recruitment of DNA repair effectors and chromatin remodeling [33]. Then the auto-PARylation on PARP1 protein causes the dissociation of PARP1 from DNA chains and restores the auto-inhibitory status of PARP1 [32].

## Clinical development of PARP inhibitors

Some nicotinamide analogs could competitively inhibit the binding of β-NAD to ART and enhance the cytotoxicity of DNA damaging agents [34]. Based on the structure of nicotinamide analogs, at least four PARPis are developed into clinical application including Olaparib, Rucaparib, Niraparib, and Talazoparib (Table 1) [35–38]. All PARPis have the capability to suppress the catalytic activity of ART [39]. However, PARPi-mediated inhibition of catalytic activity could not completely explain the phenomenon that the PARPi-mediated tumor-killing effect is greater than PARP depletion [26]. Recent studies indicated that the cytotoxicity of PARPis are more relevant to their ability of trapping PARP1 on damaged DNA chains [26]. This hypothesis is supported by the evidence that PARPis (e.g. Talazoparib) with stronger ability to trap PARP1 exhibit more potent cytotoxicity [40]. The pharmacodynamics mechanism of PARP1 is similar to topoisomerase II inhibitor which could also trap damaging repair proteins on DNA chains and induce cell death [40].

**Table 1:** Clinical PARP inhibitors

PARP inhibitor	Olaparib	Rucaparib	Niraparib	Talazoparib	Veliparib
Approvals	Ovarian cancer Breast cancer	Ovarian cancer	Ovarian cancer	Ovarian cancer	Not approved
PARP trapping potency	Middle	Middle	Middle	High	Low
Recommended dose	300 mg BID	600 mg BID	300 mg BID	1 mg QD	600 mg BID
The most common adverse reactions (in at least 20% patients)	Anemia, nausea, fatigue, vomiting, nasopharyngitis, diarrhea, arthralgia/myalgia, dysgeusia, headache, dyspepsia, decreased appetite, constipation, and stomatitis	Nausea, fatigue, vomiting, anemia, abdominal pain, dysgeusia, constipation, decreased appetite, diarrhea, thrombocytopenia, and dyspnea	Nausea, thrombocytopenia, fatigue, anemia, constipation, vomiting, neutropenia, headache, decreased appetite, insomnia, abdominal pain	Fatigue, anemia, nausea, neutropenia, headache, thrombocytopenia, vomiting, alopecia, diarrhea, decreased appetite	Nausea, fatigue

Data of Olaparib, Rucaparib, Niraparib, and Talazoparib are obtained from https://www.fda.gov/

Data of Veliparib is obtained from NCT01149083

### FDA-approved PARP inhibitors

#### Olaparib

Olaparib capsule (Lynparza, AstraZeneca) is the first PARPi entering clinical practice. Olaparib was originally approved for deleterious or suspected deleterious gBRCAm ovarian cancer patients who had undergone three or more prior lines chemotherapy [41]. Later in 2017, based on two randomized controlled trials (NCT01874353 and NCT00753545), Food and Drug Administration (FDA) approved Olaparib tablet for the maintenance treatment of recurrent epithelial ovarian, fallopian tube, or primary peritoneal cancer patients who were sensitive to platinum-based chemotherapy [42, 43]. Besides, the data of NCT01874353 supported the approval of Olaparib tablet for the maintenance treatment of gBRCAm or somatic BRCA-mutated (sBRCAm) recurrent epithelial ovarian, fallopian tube, or primary peritoneal cancer patients [42]. Then in 2018, propelled by the encouraging results of the clinical trial NCT02000622, Olaparib tablet was approved by FDA for the treatment of human epidermal growth factor receptor-2 (Her-2) negative, metastatic breast cancer patients who had been treated with chemotherapy [44]. Then in 2019, the results of a phase 3 trial (POLO study) showed that maintenance Olaparib treatment effectively prolonged the survival time of gBRCAm metastatic prostate cancer patients [45]. In this study, totally 154 patients were enrolled and the primary end-point was progression-free survival (PFS) [45]. Olaparib-treated patients had better outcomes than placebo-treated patients (the median PFS of Olaparib group vs. placebo group = 7.4 months vs. 3.8 months; Hazard Ration = 0.53; 95%CI 0.35 to 0.82; p = 0.004) [45].

#### Rucaparib

The efficacy of Rucaparib (Rubraca, Clovis Oncology Inc.) was investigated in a randomized and double-blind phase 3 clinical trial NCT01968213. This trial enrolled 564 recurrent epithelial ovarian, fallopian tube, or primary peritoneal cancer patients who had received two or more prior lines of platinum-based chemotherapy and were sensitive to the platinum-based chemotherapy [46]. The treatment effect was evaluated by PFS [46]. The results indicated that Rucaparib could effectively improve the prognosis of BRCA1/2 mutated patients (Rucaparib group vs. placebo group: Hazard Ratio = 0.23, 95% CI 0.16–0.34, p < 0.0001) [46]. Besides, analysis in all population showed that patients received Rucaparib therapy had a great advantage in PFS over placebo-treated patients: Hazard Ratio = 0.36, 95% CI = 0.30–0.45; p < 0.0001) [46]. Based on the outcome of NCT01968213, FDA approved the maintenance treatment of Rucaparib for recurrent ovarian, fallopian tube, or primary peritoneal cancer patients who were sensitive to platinum-based chemotherapy in 2018 [47].

#### Niraparib

Niraparib is developed by Tesaro which is also known as ZEJULA. In a randomized and double-blind phase 3 trial (NCT01847274), 553 platinum sensitive, recurrent ovarian cancer patients were involved [48]. In gBRCAm cohort, patients receiving Niraparib had prolonged PFS than patients treated by placebo (median PFS of Niraparib group vs. placebo group = 21.0 vs. 5.5 months, Hazard Ratio = 0.27; 95% CI 0.17–0.41, p < 0.001) [48]. In non-gBRCAm cohort, Niraparib group also exhibited better prognosis than placebo group (median PFS of Niraparib group vs. placebo group = 9.3 vs. 3.9 months, Hazard Ratio = 0.45, 95% CI 0.34–0.61, p < 0.001) [48]. This promising results of NCT01847274 directly propelled the approval of Niraparib for the maintenance treatment of platinum sensitive, recurrent epithelial ovarian, fallopian tube, or primary peritoneal cancer patients [49].

#### Talazoparib

Among all available PARPis up to now, Talazoparib (TALZENNA, Pfizer Inc.) possesses the most PARP1 trapping and cytotoxic potency. The anti-tumor effect of Talazoparib has been verified in gBRCAm advanced breast cancer patients [50]. This randomized and open-label phase 3 clinical trial (NCT01945775) recruited 431 patients [50]. Compared with standard therapy group, Talazoparib group had significantly improved outcomes (median PFS of Talazoparib group vs. standard therapy group = 8.6 vs. 5.6 months, Hazard Ratio = 0.54, 95% CI 0.41–0.71, p < 0.001) and better response rate (objective response rate of Talazoparib group vs. standard therapy group = 62.6% vs. 27.2%; Odds Ratio = 5.0; 95% CI 2.9–8.8, p < 0.001) [50]. Based on the data of NCT01945775, FDA approved Talazoparib for deleterious or suspected deleterious gBRCAm Her2‑negative, advanced breast cancer patients [51].

### PARP inhibitors in clinical trials

Until now, Veliparib has not been approved by FDA for cancer treatment. Multiple clinical studies indicated Veliparib-based combination therapy might be a promising strategy for triple negative breast cancer, ovarian cancer, pancreas ductal adenocarcinoma, myeloid leukemia, as well as hepatocellular carcinoma patients [52–56]. In a randomized, multicenter, phase 2 trial NCT01042379, total 116 stage II or III triple negative breast cancer patients received Veliparib combined Carboplatin therapy or Carboplatin monotherapy [57]. The results of NCT01042379 showed Veliparib plus Carboplatin group had a higher response rate than Carboplatin group (pathological complete response rate of Veliparib plus Carboplatin group vs. Carboplatin group: 51% vs. 26%) [57]. However, in a recent phase 3 trial NCT02032277 recruiting 634 stage II or III triple negative breast cancer patients, Veliparib plus Paclitaxel plus Carboplatin treated patients did not exhibit higher response rate than patients receiving Paclitaxel plus Carboplatin therapy (pathological complete response rate of Veliparib plus Paclitaxel plus Carboplatin group vs. Paclitaxel plus Carboplatin group: 53% vs. 58%, p = 0.36) [58]. Therefore, further investigation is needed to confirm the optimal scheme and the population which might benefit from Veliparib-based combination therapy.

## Application of PARPi beyond BRCA1/2 mutated cancer

### HR deficient cancer

BRCAness tumors are not driven by gBRCAm but share certain phenotypes. In general, BRCAness tumors harbor non-gBRCAm HR deficiency including gBRCAm and hypermethylation of BRCA1/2 promotor [59, 60]. Besides, some other core components of HR such as RAD51 recombinase (RAD51), ATM serine/threonine kinase (ATM), ATR serine/threonine kinase (ATR), partner and localizer of BRCA2 (PALB2), and Fanconi anemia gene family are determinants of intact HR as well [40, 61]. Just like gBRCAm cancer cells, cancer cells with non-gBRCAm HR deficiency exhibited the sensitivity to platinum-based drugs and PARPis [23, 62]. However, the identification of HR deficiency in cancer cells is a complex work. Thus, it is necessary to find a measurable surrogate to evaluate HR status. It has been verified that gBRCAm patients often have a specific pattern of mutations including large scale chromosomal rearrangements which might reflect impaired HR potency and hyperactive NHEJ pathway [59]. Some biomarkers such as loss of heterozygosity (LOH) closely relate with chromosomal rearrangements and predict favorable therapeutic response after PARPi treatment [63].

### Cancer with high replication stress

Even though the synergistic effect caused by PARPi and HR deficiency has been confirmed in previous studies, some HR proficient cancers such as small cell lung cancer (SCLC) showed relatively high vulnerability to PARPi [15]. This sensitivity is attributed to high replication stress driven by the loss of some tumor suppressor genes and the amplification of oncogenes [15]. SCLC possesses a unique gene expression profile which is characterized as the ubiquitous loss of TP53 and RB1 [64]. As well-studied tumor suppressor genes, TP53 and RB1 play a vital role in cell cycle checkpoint and DNA damage response [64]. Besides, the loss of RB1 abrogates the E2F1-mediated transcription inhibition of multiple DNA damage response genes including PARP1 [65]. Apart from the loss of TP53 and RB1, SCLC often harbors MYC amplification which further increases high replication stress [15]. Compared with non-small cell lung cancer (NSCLC), SCLC is more dependent on hyperactive DNA damage response and more sensitive to PARPi treatment [15].

## PARPi-involved combination therapy

In multiple PARPi-involved combination strategies, PARPi acts as sensitizers for chemotherapies, immunotherapies, and targeted therapies by limiting DNA damage repair. In addition, some targeted treatments such as MEK inhibitors could enhance the sensitivity of tumor cells and relieve the resistance to PARPi [66].

### PARPi plus genotoxic chemotherapy

Hyperactive PARP related DNA damage repair tends to result in the resistance to genotoxic chemotherapy such as Temozolomide (TMZ) and platinum compound (Table 2) [67]. Previous studies indicated that additional PARP inhibitor significantly decreased the risk of TMZ resistance and enhanced TMZ efficacy in mouse model [68–71]. These phenomena could be explained by mechanism that PARP-related DNA damage repair pathway especially BER could remove adducts from DNA chains and eliminate genotoxic chemotherapy-introduced DNA lesions [67].

**Table 2:** Clinical trials about PARP inhibitor plus chemotherapy

Combination therapy	Trial	Cancer	Phase	Status
Rucaparib and Cisplatin	NCT01074970	Breast cancer	2	Active, not recruiting
Olaparib, Paclitaxel, and Carboplatin	NCT03150576	Breast cancer	2/3	Recruiting
PF-01367338 and Carboplatin	NCT01009190	Advanced solid tumors	1	Completed
BSI-201 and Irinotecan	NCT01173497	Breast cancer	2	Completed
BSI-201, Carboplatin, and Gemcitabine	NCT00813956	Breast cancer	2	Completed
Veliparib and Topotecan Hydrochloride	NCT01012817	Multiple solid tumors	1/2	Active, not recruiting
Olaparib, Cediranib, and Platinum-based Chemotherapy	NCT02855697	Ovarian cancer	1	Recruiting
Olaparib and Platinum agents	NCT02489006	Ovarian cancer	2	Recruiting
Iniparib, Carboplatin, and Gemcitabine	NCT00540358	Breast cancer	2	Completed
AZD2281 and Liposomal Doxorubicin	NCT00628251	Ovarian cancer	2	Completed
Olaparib, Temozolomide, and Irinotecan	NCT01858168	Ewing’s sarcoma	1	Recruiting
BMN-673, Temozolomide, and Irinotecan Hydrochloride	NCT02049593	Advanced solid tumors	1	Active, not recruiting
AZD2281 and Topotecan	NCT00516438	Advanced solid tumors	1	Completed
AZD2281 and Gemcitabine	NCT00515866	Pancreatic cancer	1	Completed
AZD2281 and Dacarbazine	NCT00516802	Melanoma	1	Completed
Veliparib, VX-970, and Cisplatin	NCT02723864	Advanced solid tumors	1	Recruiting
Niraparib and Temozolomide	NCT03830918	Small cell lung cancer	1/2	Recruiting
Rucaparib and Platinum-based Chemotherapy	NCT02855944	Ovarian cancer	3	Recruiting
BGB-290 and Temozolomide	NCT03914742	Gliomas	1/2	Not yet recruiting
AZD2281, Carboplatin, and Paclitaxel	NCT00516724	Multiple solid tumors	1	Active, not recruiting
Talazoparib, Irinotecan, and Temozolomide	NCT02392793	Childhood solid tumors	1	Active, not recruiting
AZD2281, Cisplatin, and Gemcitabine	NCT00678132	Solid tumor cancers	1	Completed
Talazoparib and Temozolomide	NCT03672773	Small cell lung cancer	2	Recruiting
Veliparib and Temozolomide	NCT01139970	Acute leukemia	1	Active, not recruiting
Veliparib and Doxorubicin	NCT01145430	Ovarian cancer	1	Completed
Talazoparib and Decitabine	NCT02878785	Acute leukemia	1/2	Recruiting
Olaparib and Temozolomide	NCT03880019	Uterine leiomyosarcoma	2	Not yet recruiting
BGB-290 and Temozolomide	NCT03749187	Gliomas	1	Recruiting
Veliparib, Fluorouracil, and Irinotecan Hydrochloride	NCT02890355	Pancreatic cancer	2	Active, not recruiting
Olaparib and Temozolomide	NCT03212742	Gliomas	1/2	Recruiting
ABT-888 and Topotecan Hydrochloride	NCT00553189	Solid tumors and lymphomas	1	Completed
Olaparib and Temozolomide	NCT01390571	Glioblastoma	1	Completed
Iniparib, Gemcitabine, and Cisplatin	NCT01086254	Non-small cell lung cancer	2	Completed
Rucaparib, Docetaxel, and Carboplatin	NCT03442556	Prostate cancer	2	Recruiting
Veliparib, Carboplatin, and Paclitaxel	NCT00535119	Advanced solid cancer	1	Completed
Veliparib, Carboplatin, Paclitaxel, and Pemetrexed	NCT02944396	Non-small cell lung cancer	1/2	Active, not recruiting
Veliparib and Cyclophosphamide	NCT01351909	Breast cancer	1	Active, not recruiting
ABT-888 and Temozolomide	NCT01009788	Breast cancer	2	Active, not recruiting
BSI-201, Gemcitabine, and Carboplatin	NCT01045304	Breast cancer	2	Completed
Veliparib and Temozolomide	NCT03581292	Glioma	2	Recruiting
BSI-201, Gemcitabine, and Carboplatin	NCT01213381	Advanced solid tumors	1	Completed
Olaparib, Paclitaxel, Topotecan Hydrochloride, and Doxorubicin	NCT02502266	Ovarian cancer	2/3	Recruiting
Olaparib and Paclitaxel	NCT02789332	Breast Cancer	2	Recruiting
Veliparib, Carboplatin, Paclitaxel, and FOLFIRI	NCT02033551	Solid Tumors	1	Completed
Veliparib, Carboplatin, Cisplatin, Fluorouracil, Hydroxyurea, and Paclitaxel	NCT01711541	Head and neck cancer	1/2	Active, not recruiting
Veliparib, Gemcitabine, and Carboplatin	NCT02860819	Testicular germ cell cancer	2	Recruiting
Veliparib, Carboplatin, and Paclitaxel	NCT02264990	Non-small cell lung cancer	3	Active, not recruiting
Veliparib and Carboplatin	NCT01149083	Breast cancer	2	Active, not recruiting
Veliparib and Mitomycin C	NCT01017640	Solid tumors	1	Completed
Veliparib, Paclitaxel, and Cisplatin	NCT01281852	Cervical cancer	1	Completed
Veliparib, Paclitaxel, Carboplatin, and Bevacizumab,	NCT00989651	Ovarian cancer	1	Active, not recruiting
ABT-888 and Temozolomide	NCT00994071	Nervous system tumor	1	Completed
Veliparib and Cisplatin	NCT02595905	Breast cancer	2	Recruiting
Veliparib, Paclitaxel, and Carboplatin	NCT01366144	Solid tumors	1	Suspended
Veliparib, Gemcitabine Hydrochloride, and Cisplatin	NCT01585805	Pancreatic cancer	2	Active, not recruiting
Veliparib, Cyclophosphamide, and Doxorubicin Hydrochloride	NCT00740805	Solid tumors or non-hodgkin lymphoma	1	Active, not recruiting
Veliparib, Topotecan Hydrochloride, and Carboplatin	NCT00588991	Acute leukemia, high-risk myelodysplasia, and myeloproliferative disorders	1	Active, not recruiting
Veliparib, Bendamustine Hydrochloride, and Rituximab	NCT01326702	Lymphoma, multiple myeloma, solid tumors	1/2	Completed
Veliparib, Cisplatin, and Vinorelbine Ditartrate	NCT01104259	Breast cancer	1	Completed

The details of the table are obtained from https://www.clinicaltrials.gov/

#### PARPi plus TMZ

As a widely adopted DNA-alkylating agent, TMZ could spontaneously hydrolyze and release reactive methyldiazonium ion which eventually leads to the production of DNA adducts [72]. PARPi is regard as an effective sensitizer for TMZ by counteracting the PARP-BER-mediated detoxification [73]. Hussain et al. conducted a single-arm phase 1 trial (NCT01085422) to evaluate the safety and efficacy of low dose Veliparib plus TMZ combination therapy in metastatic castration-resistant prostate cancer patients [74]. The results showed this combination therapy was well-tolerant while its anti-cancer effect was relative modest (Just 3 out of 25 patients showed confirmed PSA response) [74]. Nevertheless, more clinical trials exploring the effect of PARPi and TMZ are ongoing [75].

#### PARPi plus platinum

Similar to TMZ, platinum compounds could also generate adducts to DNA chains which leads to the formation of stable intra-strand cross-links [76]. As a result, the replication and transcription processes in treated cells are severely interfered. Platinum resistance is closely related with DNA damage repair and could be overcome by PARPi [77]. In preclinical experiment, Olaparib and Veliparib remarkably potentiated cisplatin-induced cytotoxicity [78]. Later, a phase 2 study (NCT01081951) assessed the efficacy of the combination therapy of Olaparib plus platinum-based chemotherapy in platinum-sensitive, recurrent ovarian cancer patients [79, 80]. The results demonstrated that patients receiving Olaparib plus platinum-based chemotherapy had markedly better outcomes than chemotherapy treated patients (median overall survival of Olaparib plus platinum-based chemotherapy group vs. chemotherapy group: 12.2 vs 9.6 months, Hazard Ratio = 0.51, 95% CI 0.34–0.77, p = 0.0012) [79]. In 2018, Loibl et al. reported the results of phase 3 trial (NCT02032277) which evaluated the efficacy of Veliparib plus carboplatin plus paclitaxel combination therapy in triple-negative breast cancer patients [58]. Patients undergoing concurrent Veliparib plus carboplatin plus paclitaxel had a significantly increased response rate than paclitaxel-treated patients (53% vs. 31%, p < 0.0001) [58].

### PARPi plus immune checkpoint inhibitor

As the crucial co-inhibitory molecules regulating immune activation and tolerance, programmed cell death-1 (PD-1) and cytotoxic T-lymphocyte-associated protein 4 (CTLA-4) induce dephosphorylation via intracellular immunoreceptor tyrosine-based inhibitory motif (ITIM) [81–83]. T cell receptor (TCR) mediated tyrosine phosphorylation and T cell activation are undermined [84]. In tumor microenvironment, the expression of PD-L1 is usually upregulated which increases the ratio of exhausted T cells and interferes robust immune surveillance [85]. Immune checkpoint inhibitors (ICI) restore T cell from exhausted status and stimulate anti-cancer immune response [86]. However, the clinical application of ICI is limited by low response rate which is related with tumor mutation burden and the status of tumor infiltrating lymphocytes (TILs) [87, 88].

PARPi therapy has a substantial influence on systemic immune response [89]. On the one hand, PARPi introduces large scale chromosome recombination which might generate quantities of neoantigen and increase the immunogenicity of cancers [40]. On the other hand, PARPi-induced DSB could be detected by cytosolic DNA sensor and activates the downstream cyclic GMP-AMP synthase (cGAS)-stimulator of interferon genes (STING)-type-I interferon (IFN) pathway [90]. Type I IFN is a versatile molecule which promotes the cross-presentation of dendritic cell (DC), enhances the trafficking and migration of T cells, as well as induces the secretion of Th1-skewing cytokines [91, 92]. Compared with low level inflammation in baseline, PARPi treatment leads to catastrophic DNA damage and acute inflammation [89]. This PARPi-introduced transformation of microenvironment facilitates immune priming and activation [89]. In mouse model bearing SCLC, combination therapy of Olaparib and anti-PD-L1 showed more potent anti-cancer effect than monotherapy and induced complete tumor regression in all treated mice [90]. Immune profiling of resected tumors indicated that the combination therapy significantly elevated the abundance of tumor infiltrating CD3^+^ T cells and CD8^+^ cytotoxic T cells [90]. Moreover, the synergistic effect between PARPi and ICI was confirmed in multiple mice cancer models including breast cancer, ovarian carcinoma, and skin tumor [93, 94]. Accumulating evidence demonstrated that PARPi could promote anti-cancer immune response while anti-PD-1/PD-L1 could neutralize PARPi-induced PD-L1 upregulation [89].

In 2018, Karzai et al. reported the results of phase 2 clinical trial NCT02484404. 17 metastatic castration-resistant prostate cancer patients were enrolled into this study and received Olaparib plus Durvalumab treatment [95]. The results showed that the toxicity of combination therapy was acceptable (grade 3/4 adverse event occurred in 2/17 patients) and the efficacy of combination therapy was satisfactory especially in DNA damage repair deficient patients (median PFS: 16.1 months, 95% CI 7.8–18.1 months) [95]. The efficacy of combination scheme of Olaparib plus Durvalumab was also evaluated in relapsed SCLC patients [96]. The results of SCLC cohort of NCT02484404 showed that the response rate of overall SCLC patients was relative low (Ration of patients with confirmed responses or prolonged stable disease: 21.1%; 95% CI 6.1–45.6%), but all patients with tumors classified as inflamed phenotype exhibited positive therapeutic response [96].

### PARPi plus targeted therapy

Acquired resistance to PARPi is an important obstacle which has not been well resolved. Factors such as secondary reversion BRCA1/2 mutation, loss of PAPR1, as well as restoration of HR are related to PARPi resistance. Oncogene-related signaling pathways such as androgen receptor (AR), mitogen-activated protein kinase (MEK), BET bromodomain (BRD4) pathways could directly drive the expression of HR related proteins and induce PARPi resistance [15].

#### PARPi plus AR inhibitor

Asim et al. found that intact AR signaling was indispensable to maintain the expression and activity of HR related genes in prostate cancer cells [97]. After androgen-deprivation therapy, the activity of HR was impaired and prostate cancer cell was highly dependent on PARP-BER pathway to repair DNA damages [97]. This artificially induced BRCAness phenotype endows the sensitivity to PARPi treatment in prostate cancer cells [98]. In 2018, a phase 2 trial (NCT01972217) confirmed the efficacy of double blockade of AR and PARP in metastatic, castration resistant prostate cancer patients [99]. Patients receiving Olaparib plus Abiraterone therapy had better survival data than patients treated with Abiraterone therapy (median radiographic PFS of Olaparib plus Abiraterone group vs. Abiraterone group: 3.8 vs. 8.2 months, Hazard ratio = 0.65, 95% CI 0.44–0.97, p = 0.034) [99].

#### PARPi plus MEK inhibitor

Sun et al. found that MEK inhibitor could increase the sensitivity to PARPi treatment in RAS mutated ovarian cancer patients by inhibiting HR repair activity and elevating PARP expression [100]. Besides, PARPi plus MEK inhibitor therapy induced cell apoptosis by activating BIM signaling [100]. This MEK inhibitor-based combination therapy showed potent anti-cancer effect in multiple cancer cell lines and mice models not limited to BRCA1/2 mutated cells [100]. The results of in vivo and in vitro experiments showed that the combination strategy is a promising manner to overcome PARPi resistance and increase the response intensity, duration, and spectrum of PARPi.

#### PARPi plus BRD4 inhibitor

BRD4 promotes cancer cell proliferation and survival by maintaining and facilitating oncogenic transcription [101]. The expression of BRD4 is often upregulated and predicts poor prognosis in high-grade serous ovarian carcinoma patients [102]. Sun et al. found that BRD4 bound to the promoter and enhancer of C-terminal binding protein interacting protein (CtIP) which was the core component of HR pathway [103]. BRD4 inhibitor suppressed the expression of CtIP and interfered the recruitment of DNA damage repair proteins to DNA lesions [103]. In vitro experiments, BRD4 inhibitor treatment restored the sensitivity of to PARPi therapy in PARPi-resistant cells [103]. In vivo experiment, the combination therapy of PARPi and BRD4 inhibitor effectively prolonged tumor control in multiple patient-derived tumor xenograft models including HR proficient ovarian and breast cancers [103].

## Conclusion

Synthetic lethal interaction is context-dependent where the alteration in first gene leads to the essential role of second gene for the viability of cancer cells. Targeting the product of second gene could selectively kill malignant cells with minor effect on nonmalignant cells. Since synthetic lethal effect was proposed nearly 100 years ago, this hypothesis has been intensively studied. PARPi is the first agent based on synthetic lethal concept. The great success of PARPi in preclinical and clinical studies propels the approval of four PARPis for BRCA1/2 mutated ovarian and breast cancer patients. However, data of some clinical trials showed that a broader range of populations might benefit from PARPi. Establishing a comprehensive evaluation framework to select candidates for PARPi treatment is necessary. Besides, combination therapy with additional ICI, HR targeting agents, as well as chemotherapy have shown synergistic effect even in PARPi resistant models. Accumulating evidence in preclinical studies indicates PARPi is a promising therapy cross multiple cancer types. We believe the future clinical studies would provide more novel perspectives for optimal PARPi-based combination scheme.

## Data Availability

Data sharing not applicable to this article as no datasets were generated or analyzed during the current study.

## Abbreviations

SSB: DNA single-strand break
DSB: double-strand break
MMR: mismatch repair
NER: nucleotide excision repair
BER: base excision repair
HR: homologous recombination
NHEJ: nonhomologous end joining
PARP: Poly (ADP-ribose) polymerase
gBRCAm: germline BRCA1/2 mutated
HD: helical domain
ART: ADP-ribosyltransferase catalytic domain
β-NAD: β-nicotinamide adenine dinucleotide
FDA: Food and Drug Administration
sBRCAm: somatic BRCA-mutated
PFS: progression-free survival
LOH: loss of heterozygosity
SCLC: small cell lung cancer
NSCLC: non-small cell lung cancer
TMZ: Temozolomide
PD-1: programmed cell death-1
CTLA-4: cytotoxic T-lymphocyte-associated protein 4
ITIM: intracellular immunoreceptor tyrosine-based inhibitory motif
TCR: T cell receptor
ICI: immune checkpoint inhibitors
TIL: tumor infiltrating lymphocyte
cGAS: cyclic GMP-AMP synthase
STING: stimulator of interferon genes
IFN: interferon
DC: dendritic cell
AR: androgen receptor
MEK: mitogen-activated protein kinase
BRD4: BET bromodomain
CtIP C: C-terminal binding protein interacting protein

## References

[1] Ben-David U, Beroukhim R, Golub TR (2019). Genomic evolution of cancer models: perils and opportunities. Nat Rev Cancer.

[2] Kalimutho M, Nones K, Srihari S (2019). Patterns of genomic instability in breast cancer. Trends Pharmacol Sci.

[3] Yi M, Qin S, Zhao W (2018). The role of neoantigen in immune checkpoint blockade therapy. Exp Hematol Oncol..

[4] Marin-Acevedo JA, Soyano AE, Dholaria B, Knutson KL, Lou Y (2018). Cancer immunotherapy beyond immune checkpoint inhibitors. J Hematol Oncol..

[5] Motegi A, Masutani M, Yoshioka KI, Bessho T (2019). Aberrations in DNA repair pathways in cancer and therapeutic significances. Semin Cancer Biol.

[6] Hu H, Li H, Jiao F (2017). Association of a novel point mutation in MSH2 gene with familial multiple primary cancers. J Hematol Oncol..

[7] Lord CJ, Tutt AN, Ashworth A (2015). Synthetic lethality and cancer therapy: lessons learned from the development of PARP inhibitors. Annu Rev Med.

[8] Hu X, Huang W, Fan M (2017). Emerging therapies for breast cancer. J Hematol Oncol..

[9] George A, Kaye S, Banerjee S (2017). Delivering widespread BRCA testing and PARP inhibition to patients with ovarian cancer. Nat Rev Clin Oncol..

[10] Jalal S, Earley JN, Turchi JJ (2011). DNA repair: from genome maintenance to biomarker and therapeutic target. Clin Cancer Res.

[11] di Ghelli Luserna Rora A, Iacobucci I, Martinelli G (2017). The cell cycle checkpoint inhibitors in the treatment of leukemias. J Hematol Oncol..

[12] Germano G, Amirouchene-Angelozzi N, Rospo G, Bardelli A (2018). The clinical impact of the genomic landscape of mismatch repair-deficient cancers. Cancer Discov.

[13] Zhu J, Jia W, Wu C (2018). Base excision repair gene polymorphisms and wilms tumor susceptibility. EBioMedicine..

[14] Amir E, Seruga B, Serrano R, Ocana A (2010). Targeting DNA repair in breast cancer: a clinical and translational update. Cancer Treat Rev.

[15] Pilie PG, Gay CM, Byers LA, O’Connor MJ, Yap TA (2019). PARP inhibitors: extending benefit beyond BRCA-Mutant cancers. Clin Cancer Res.

[16] Kaniecki K, De Tullio L, Greene EC (2018). A change of view: homologous recombination at single-molecule resolution. Nat Rev Genet.

[17] Balmus G, Pilger D, Coates J (2019). ATM orchestrates the DNA-damage response to counter toxic non-homologous end-joining at broken replication forks. Nat Commun..

[18] Metzger MJ, Stoddard BL, Monnat RJ (2013). PARP-mediated repair, homologous recombination, and back-up non-homologous end joining-like repair of single-strand nicks. DNA Repair.

[19] Aparicio T, Baer R, Gautier J (2014). DNA double-strand break repair pathway choice and cancer. DNA Repair.

[20] Mengwasser KE, Adeyemi RO, Leng Y (2019). Genetic screens reveal FEN1 and APEX2 as BRCA2 synthetic lethal targets. Mol Cell.

[21] Groschel S, Hubschmann D, Raimondi F (2019). Defective homologous recombination DNA repair as therapeutic target in advanced chordoma. Nat Commun..

[22] Fong PC, Boss DS, Yap TA (2009). Inhibition of poly(ADP-ribose) polymerase in tumors from BRCA mutation carriers. N Engl J Med.

[23] Fong PC, Yap TA, Boss DS (2010). Poly(ADP)-ribose polymerase inhibition: frequent durable responses in BRCA carrier ovarian cancer correlating with platinum-free interval. J Clin Oncol.

[24] Mukhopadhyay A, Plummer ER, Elattar A (2012). Clinicopathological features of homologous recombination-deficient epithelial ovarian cancers: sensitivity to PARP inhibitors, platinum, and survival. Cancer Res.

[25] Helleday T (2011). The underlying mechanism for the PARP and BRCA synthetic lethality: clearing up the misunderstandings. Mol Oncol..

[26] Murai J, Huang SY, Das BB (2012). Trapping of PARP1 and PARP2 by clinical PARP inhibitors. Cancer Res.

[27] Farmer H, McCabe N, Lord CJ (2005). Targeting the DNA repair defect in BRCA mutant cells as a therapeutic strategy. Nature.

[28] Keung MYT, Wu Y, Vadgama JV (2019). PARP inhibitors as a therapeutic agent for homologous recombination deficiency in breast cancers. J Clin Med..

[29] Faraoni I, Graziani G (2018). Role of BRCA mutations in cancer treatment with poly(ADP-ribose) polymerase (PARP) inhibitors. Cancers (Basel)..

[30] Krishnakumar R, Kraus WL (2010). The PARP side of the nucleus: molecular actions, physiological outcomes, and clinical targets. Mol Cell.

[31] Murai J, Huang SY, Renaud A (2014). Stereospecific PARP trapping by BMN 673 and comparison with olaparib and rucaparib. Mol Cancer Ther.

[32] Rouleau M, Patel A, Hendzel MJ, Kaufmann SH, Poirier GG (2010). PARP inhibition: PARP1 and beyond. Nat Rev Cancer.

[33] Schreiber V, Dantzer F, Ame JC, de Murcia G (2006). Poly(ADP-ribose): novel functions for an old molecule. Nat Rev Mol Cell Biol.

[34] Terada M, Fujiki H, Marks PA, Sugimura T (1979). Induction of erythroid differentiation of murine erythroleukemia cells by nicotinamide and related compounds. Proc Natl Acad Sci USA..

[35] Kaufman B, Shapira-Frommer R, Schmutzler RK (2015). Olaparib monotherapy in patients with advanced cancer and a germline BRCA1/2 mutation. J Clin Oncol.

[36] Swisher EM, Lin KK, Oza AM (2017). Rucaparib in relapsed, platinum-sensitive high-grade ovarian carcinoma (ARIEL2 Part 1): an international, multicentre, open-label, phase 2 trial. Lancet Oncol..

[37] Sandhu SK, Schelman WR, Wilding G (2013). The poly(ADP-ribose) polymerase inhibitor niraparib (MK4827) in BRCA mutation carriers and patients with sporadic cancer: a phase 1 dose-escalation trial. Lancet Oncol..

[38] de Bono J, Ramanathan RK, Mina L (2017). Phase I, dose-escalation, two-part trial of the PARP inhibitor talazoparib in patients with advanced germline BRCA1/2 mutations and selected sporadic cancers. Cancer Discov.

[39] Thomas A, Murai J, Pommier Y (2018). The evolving landscape of predictive biomarkers of response to PARP inhibitors. J Clin Invest..

[40] Lord CJ, Ashworth A (2017). PARP inhibitors: Synthetic lethality in the clinic. Science.

[41] Liu FW, Tewari KS (2016). New targeted agents in gynecologic cancers: synthetic lethality, homologous recombination deficiency, and PARP inhibitors. Curr Treat Options Oncol.

[42] Friedlander M, Gebski V, Gibbs E (2018). Health-related quality of life and patient-centred outcomes with olaparib maintenance after chemotherapy in patients with platinum-sensitive, relapsed ovarian cancer and a BRCA1/2 mutation (SOLO2/ENGOT Ov-21): a placebo-controlled, phase 3 randomised trial. Lancet Oncol..

[43] Ledermann JA, Harter P, Gourley C (2016). Overall survival in patients with platinum-sensitive recurrent serous ovarian cancer receiving olaparib maintenance monotherapy: an updated analysis from a randomised, placebo-controlled, double-blind, phase 2 trial. Lancet Oncol..

[44] Robson M, Im SA, Senkus E (2017). Olaparib for metastatic breast cancer in patients with a germline BRCA mutation. N Engl J Med.

[45] Golan T, Hammel P, Reni M (2019). Maintenance olaparib for germline BRCA-mutated metastatic pancreatic cancer. N Engl J Med.

[46] Coleman RL, Oza AM, Lorusso D (2017). Rucaparib maintenance treatment for recurrent ovarian carcinoma after response to platinum therapy (ARIEL3): a randomised, double-blind, placebo-controlled, phase 3 trial. Lancet.

[47] Pearre DC, Tewari KS (2018). Targeted treatment of advanced ovarian cancer: spotlight on rucaparib. Ther Clin Risk Manag.

[48] Mirza MR, Monk BJ, Herrstedt J (2016). Niraparib maintenance therapy in platinum-sensitive, recurrent ovarian cancer. N Engl J Med.

[49] Ethier JL, Lheureux S, Oza AM (2018). The role of niraparib for the treatment of ovarian cancer. Future Oncol..

[50] Litton JK, Rugo HS, Ettl J (2018). Talazoparib in patients with advanced breast cancer and a germline BRCA mutation. N Engl J Med.

[51] McCann KE (2019). Advances in the use of PARP inhibitors for BRCA1/2-associated breast cancer: talazoparib. Future Oncol..

[52] Lowery MA, Kelsen DP, Capanu M (2018). Phase II trial of veliparib in patients with previously treated BRCA-mutated pancreas ductal adenocarcinoma. Eur J Cancer.

[53] Gojo I, Beumer JH, Pratz KW (2017). A phase 1 study of the PARP inhibitor veliparib in combination with temozolomide in acute myeloid leukemia. Clin Cancer Res.

[54] Rodler ET, Kurland BF, Griffin M (2016). Phase I study of veliparib (ABT-888) combined with cisplatin and vinorelbine in advanced triple-negative breast cancer and/or BRCA mutation-associated breast cancer. Clin Cancer Res.

[55] Gabrielson A, Tesfaye AA, Marshall JL (2015). Phase II study of temozolomide and veliparib combination therapy for sorafenib-refractory advanced hepatocellular carcinoma. Cancer Chemother Pharmacol.

[56] Gray HJ, Bell-McGuinn K, Fleming GF (2018). Phase I combination study of the PARP inhibitor veliparib plus carboplatin and gemcitabine in patients with advanced ovarian cancer and other solid malignancies. Gynecol Oncol.

[57] Rugo HS, Olopade OI, DeMichele A (2016). Adaptive randomization of veliparib-carboplatin treatment in breast cancer. N Engl J Med.

[58] Loibl S, O’Shaughnessy J, Untch M (2018). Addition of the PARP inhibitor veliparib plus carboplatin or carboplatin alone to standard neoadjuvant chemotherapy in triple-negative breast cancer (BrighTNess): a randomised, phase 3 trial. Lancet Oncol..

[59] Lord CJ, Ashworth A (2016). BRCAness revisited. Nat Rev Cancer.

[60] Timms KM, Abkevich V, Hughes E (2014). Association of BRCA1/2 defects with genomic scores predictive of DNA damage repair deficiency among breast cancer subtypes. Breast Cancer Res.

[61] Turner N, Tutt A, Ashworth A (2004). Hallmarks of ‘BRCAness’ in sporadic cancers. Nat Rev Cancer.

[62] McCabe N, Turner NC, Lord CJ (2006). Deficiency in the repair of DNA damage by homologous recombination and sensitivity to poly(ADP-ribose) polymerase inhibition. Cancer Res.

[63] Watkins JA, Irshad S, Grigoriadis A, Tutt AN (2014). Genomic scars as biomarkers of homologous recombination deficiency and drug response in breast and ovarian cancers. Breast Cancer Res.

[64] George J, Lim JS, Jang SJ (2015). Comprehensive genomic profiles of small cell lung cancer. Nature.

[65] Byers LA, Wang J, Nilsson MB (2012). Proteomic profiling identifies dysregulated pathways in small cell lung cancer and novel therapeutic targets including PARP1. Cancer Discov.

[66] Shao N, Shi Y, Yu L (2019). Prospect for Application of PARP Inhibitor in Patients with HER2 Negative Breast Cancer. Int J Biol Sci..

[67] Lu Y, Liu Y, Pang Y, Pacak K, Yang C (2018). Double-barreled gun: combination of PARP inhibitor with conventional chemotherapy. Pharmacol Ther.

[68] Cheng CL, Johnson SP, Keir ST (2005). Poly(ADP-ribose) polymerase-1 inhibition reverses temozolomide resistance in a DNA mismatch repair-deficient malignant glioma xenograft. Mol Cancer Ther.

[69] Tentori L, Leonetti C, Scarsella M (2002). Combined treatment with temozolomide and poly(ADP-ribose) polymerase inhibitor enhances survival of mice bearing hematologic malignancy at the central nervous system site. Blood.

[70] Smith MA, Reynolds CP, Kang MH (2015). Synergistic activity of PARP inhibition by talazoparib (BMN 673) with temozolomide in pediatric cancer models in the pediatric preclinical testing program. Clin Cancer Res.

[71] Gupta SK, Kizilbash SH, Carlson BL (2016). Delineation of MGMT hypermethylation as a biomarker for veliparib-mediated temozolomide-sensitizing therapy of glioblastoma. J Natl Cancer Inst.

[72] Schreck KC, Grossman SA (2018). Role of temozolomide in the treatment of cancers involving the central nervous system. Oncology.

[73] Sarkaria JN, Kitange GJ, James CD (2008). Mechanisms of chemoresistance to alkylating agents in malignant glioma. Clin Cancer Res.

[74] Hussain M, Carducci MA, Slovin S (2014). Targeting DNA repair with combination veliparib (ABT-888) and temozolomide in patients with metastatic castration-resistant prostate cancer. Invest New Drugs.

[75] Lesueur P, Lequesne J, Grellard JM (2019). Phase I/IIa study of concomitant radiotherapy with olaparib and temozolomide in unresectable or partially resectable glioblastoma: OLA-TMZ-RTE-01 trial protocol. BMC Cancer..

[76] Lazarevic T, Rilak A, Bugarcic ZD (2017). Platinum, palladium, gold and ruthenium complexes as anticancer agents: current clinical uses, cytotoxicity studies and future perspectives. Eur J Med Chem.

[77] Nguewa PA, Fuertes MA, Cepeda V (2006). Poly(ADP-ribose) polymerase-1 inhibitor 3-aminobenzamide enhances apoptosis induction by platinum complexes in cisplatin-resistant tumor cells. Med Chem.

[78] Cheng H, Zhang Z, Borczuk A (2013). PARP inhibition selectively increases sensitivity to cisplatin in ERCC1-low non-small cell lung cancer cells. Carcinogenesis.

[79] Oza AM, Cibula D, Benzaquen AO (2015). Olaparib combined with chemotherapy for recurrent platinum-sensitive ovarian cancer: a randomised phase 2 trial. Lancet Oncol..

[80] Gunderson CC, Moore KN (2015). PARP inhibition in ovarian cancer: state of the science. Gynecol Oncol.

[81] Yi M, Yu S, Qin S (2018). Gut microbiome modulates efficacy of immune checkpoint inhibitors. J Hematol Oncol..

[82] Yi M, Jiao D, Xu H (2018). Biomarkers for predicting efficacy of PD-1/PD-L1 inhibitors. Mol Cancer..

[83] Long J, Lin J, Wang A (2017). PD-1/PD-L blockade in gastrointestinal cancers: lessons learned and the road toward precision immunotherapy. J Hematol Oncol..

[84] Bardhan K, Anagnostou T, Boussiotis VA (2016). The PD1:PD-L1/2 pathway from discovery to clinical implementation. Front Immunol..

[85] Ok CY, Young KH (2017). Checkpoint inhibitors in hematological malignancies. J Hematol Oncol..

[86] Yang J, Hu L (2019). Immunomodulators targeting the PD-1/PD-L1 protein-protein interaction: from antibodies to small molecules. Med Res Rev.

[87] Li X, Shao C, Shi Y, Han W (2018). Lessons learned from the blockade of immune checkpoints in cancer immunotherapy. J Hematol Oncol..

[88] Yi M, Jiao D, Qin S (2019). Synergistic effect of immune checkpoint blockade and anti-angiogenesis in cancer treatment. Mol Cancer..

[89] Stewart RA, Pilie PG, Yap TA (2018). Development of PARP and immune-checkpoint inhibitor combinations. Cancer Res.

[90] Sen T, Rodriguez BL, Chen L (2019). Targeting DNA Damage response promotes antitumor immunity through STING-mediated T-cell activation in small cell lung cancer. Cancer Discov.

[91] Zitvogel L, Galluzzi L, Kepp O, Smyth MJ, Kroemer G (2015). Type I interferons in anticancer immunity. Nat Rev Immunol.

[92] Li A, Yi M, Qin S (2019). Activating cGAS-STING pathway for the optimal effect of cancer immunotherapy. J Hematol Oncol..

[93] Jiao S, Xia W, Yamaguchi H (2017). PARP inhibitor upregulates PD-L1 expression and enhances cancer-associated immunosuppression. Clin Cancer Res.

[94] Wang Z, Sun K, Xiao Y (2019). Niraparib activates interferon signaling and potentiates anti-PD-1 antibody efficacy in tumor models. Sci Rep..

[95] Karzai F, VanderWeele D, Madan RA (2018). Activity of durvalumab plus olaparib in metastatic castration-resistant prostate cancer in men with and without DNA damage repair mutations. J Immunother Cancer..

[96] Thomas A, Vilimas R, Trindade C (2019). Durvalumab in combination with olaparib in patients with relapsed small cell lung cancer: results from a phase II study. J Thorac Oncol..

[97] Asim M, Tarish F, Zecchini HI (2017). Synthetic lethality between androgen receptor signalling and the PARP pathway in prostate cancer. Nat Commun..

[98] Li L, Karanika S, Yang G (2017). Androgen receptor inhibitor-induced “BRCAness” and PARP inhibition are synthetically lethal for castration-resistant prostate cancer. Sci Signal..

[99] Clarke N, Wiechno P, Alekseev B (2018). Olaparib combined with abiraterone in patients with metastatic castration-resistant prostate cancer: a randomised, double-blind, placebo-controlled, phase 2 trial. Lancet Oncol..

[100] Sun C, Fang Y, Yin J (2017). Rational combination therapy with PARP and MEK inhibitors capitalizes on therapeutic liabilities in RAS mutant cancers. Sci Transl Med..

[101] Loven J, Hoke HA, Lin CY (2013). Selective inhibition of tumor oncogenes by disruption of super-enhancers. Cell.

[102] Zhang Z, Ma P, Jing Y (2016). BET bromodomain inhibition as a therapeutic strategy in ovarian cancer by downregulating FoxM1. Theranostics..

[103] Sun C, Yin J, Fang Y (2018). BRD4 inhibition is synthetic lethal with PARP inhibitors through the induction of homologous recombination deficiency. Cancer Cell.

[104] Chen X, Huan X, Liu Q (2018). Design and synthesis of 2-(4,5,6,7-tetrahydrothienopyridin-2-yl)-benzoimidazole carboxamides as novel orally efficacious Poly(ADP-ribose)polymerase (PARP) inhibitors. Eur J Med Chem.

